# Risk Factors for Abdominal Aortic Aneurysm in Population-Based Studies: A Systematic Review and Meta-Analysis

**DOI:** 10.3390/ijerph15122805

**Published:** 2018-12-10

**Authors:** Emma Altobelli, Leonardo Rapacchietta, Valerio F. Profeta, Roberto Fagnano

**Affiliations:** 1 Department of Life, Health and Environmental Sciences, University of L’Aquila, 67100 L’Aquila, Italy; 2 Epidemiology and Biostatistics Unit, Local Health Unit, 64100 Teramo, Italy; leonardo.rapacchietta@gmail.com; 3 Department of community Health, Local Health Unit, 64100 Teramo, Italy; valerio.profeta@aslteramo.it; 4 Manager of Local Health Unit 4, 64100 Teramo, Italy; roberto.fagnano@aslteramo.it

**Keywords:** abdominal aortic aneurysm, risk factors, observational studies, meta-analysis

## Abstract

Abdominal aortic aneurysm (AAA) represents an important public health problem with a prevalence between 1.3% and 12.5%. Several population-based randomized trials have evaluated ultrasound screening for AAA providing evidence of a reduction in aneurysm-related mortality in the screened population. The aim of our study was to perform a systematic review and meta-analysis of the risk factors for AAA. We conducted a systematic review of observational studies and we performed a meta-analysis that evaluated the following risk factors: gender, smoking habits, hypertension, coronary artery disease and family history of AAA. Respect to a previous a meta-analysis we added the funnel plot to examine the effect sizes estimated from individual studies as measure of their precision; sensitivity analysis to check the stability of study findings and estimate how the overall effect size would be modified by removal of one study; cumulative analysis to evaluate the trend between studies in relation to publication year. Abdominal aortic aneurysm prevalence is higher in smokers and in males. On the other hand, while diabetes is a risk factor for many cardiovascular diseases, it is not a risk factor for AAA. In addition, it is important to underline that all countries, where AAA screening was set up, had high income level and the majority belong to Western Europe (United Kingdom, Sweden, Italy, Poland, Spain and Belgium). Abdominal aortic aneurysm screening is fundamental for public health. It could avoid deaths, ruptures, and emergency surgical interventions if abdominal aortic aneurysm was diagnosed early in the population target for screening.

## 1. Introduction

Abdominal aortic aneurysm (AAA) is defined as a permanent dilation of the abdominal aorta, with a diameter of 3 cm or more [1], that generally remains asymptomatic until its rupture. It is the result of a loss of elastic lamina and smooth muscle cells, which could be due to inflammatory agents and matrix metalloproteases [2].

Abdominal aortic aneurysm represents an important public health problem with a prevalence between 1.3% and 12.5% in males, and between 0.0% and 5.2% in females [3]. In women, it generally appears 10 years later than in males [4]. Abdominal aortic aneurysm represents about 1% of deaths in males over the age of 65, causing more than 175,000 deaths worldwide [5]. The mortality rate associated with rupture is very high and varies between 60% and 80%, early diagnosis and treatment therefore is very important before its rupture [6]. Rupture rates increase markedly with aneurysm diameter; for each 0.5 cm increase in AAA diameter, rates increase by 0.5 mm/year and rupture rates double [7]. Other more frequent risk factors associated with AAA are: age, gender, hypertension, family history and coronary artery disease [8]. Although the role of hypertension is still controversial [9,10,11,12], it is considered a risk factor in some studies [13,14,15].

Diabetes is a risk factor for many cardiovascular diseases, it is not a risk factor for AAA. On the contrary, it is negatively associated with AAA. This evidence could be associated to therapy with metformin [16,17,18,19]. Moreover, it is important to underline that the presence of AAA in a part of the population remains unexplained and other risk factors may be involved as well as an interaction between genetic and epigenetic background [20,21]. Abdominal aorta aneurysm can be easily diagnosed using ultrasound, a simple highly reliable non-invasive reproductive method. Intervention at this stage could reduce the frequency of rupture, reduce mortality and the requirement for emergency hospital treatment.

Several population-based randomized trials have evaluated ultrasound screening for AAA providing evidence of a reduction in aneurysm-related mortality in the screened population [22,23,24,25]. Thompson et al. showed the mortality benefit of screening men aged 65–74 for abdominal aortic aneurysm is maintained up to 10 years and cost effectiveness becomes more favorable over time [26]. 

Furthermore, it has been recently shown that the implementation of a screening system reduces not only costs, but has many benefits in terms of life expectancy [27]. Based on this evidence, the World Health Organization has included AAA screening among the interventions that proved to be cost effective. Despite this, only a few developed countries in the world have set up screening programmes for AAA [28]. At present, US Preventive Service Task Force (USPSTF) guidelines [29], have strongly recommended a one-time AAA screening for men aged 65–75 who have smoked. The aim of our study was to perform a systematic review and meta-analysis of the literature of the observational studies that evaluated the presence of the following determinants: gender, smoking habits, hypertension, diabetes mellitus, coronary artery disease (CAD) and family history of AAA.

## 2. Material and Methods

### 2.1. Search Method for Identification of Studies

The papers to be included in this systematic review and meta-analysis were sought in the MEDLINE, EMBASE, Scopus, Clinicaltrials.gov, Web of Science, and Cochrane Library databases up to 30 June 2018. The search strategy was conducted using the following terms: Abdominal Aortic Aneurysms OR Aneurysms, Abdominal Aortic OR Aortic Aneurysms, Abdominal OR Abdominal Aortic Aneurysm OR Aneurysm, Abdominal Aortic AND Screening OR Mass Screenings OR Screening, Mass OR Screenings, Mass OR Screenings AND Factor, Risk OR Factors, Risk OR Risk Factor OR Population at Risk OR Risk, Population at OR Populations at Risk OR Risk, Populations at NOT surgical repair. The period considered was 30 June 1990–1 June 2018. Only papers written in English language were considered.

**Figure 1:**
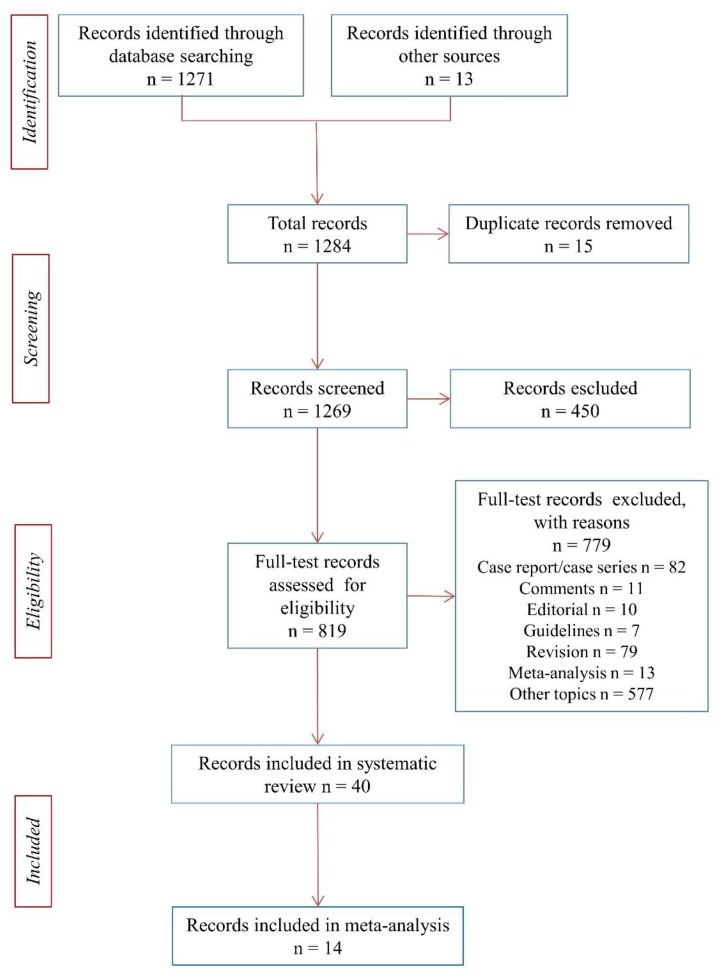
Flow chart of search strategy.

The methodology used is described in Preferred Reporting Items for Systematic reviews and Meta-Analyses (PRISMA) Flow-Chart (Figure 1) [30].

### 2.2. Criteria for Selecting Studies

Determinants as gender, smoking habits, hypertension, diabetes mellitus, family history of AAA and CAD were considered in our Meta-Analysis. All publication years and only English language papers are included in a specific table (Table 1). Observational studies were included in the meta-analysis.

#### 2.2.1. Participants

Males and females were considered together. The age of the population target included in our Meta-Analysis varies according to each study. Therefore, a unique range cannot be defined.

#### 2.2.2. Outcome

Abdominal Aortic Aneurysm is a pathology diagnosed when abdominal aorta has a diameter of 3 cm or more. We included studies that evaluated the potential risk factors associated to AAA and described it above in the eligibility criteria and study design section.

#### 2.2.3. Quality Assessment

The papers were selected by two independent reviewers (V.F.P and L.R.); a methodologist (E.A.) resolved any disagreements.

### 2.3. Statistical Analysis

Meta-Analyses were performed when there were at least four studies. Odds ratios (ORs) with 95% CI and *p*-value was used as a measure of effect size. A random effect model was applied as a conservative approach to account for different sources of variation among studies. Heterogeneity was assessed using Q statistics and *I*^2^. Publication bias analysis was estimated using Egger’s linear regression test [31], Begg’s test [31] and Mazumdar’s rank correlation test [32]. The trim and fill procedure was used to check the publication. Finally, meta-regression analyses were performed using publication year as a moderator variable (random effect model) where appropriate.

Prometa 3 (Internovi, Cesena, Italy) was used for all statistical analysis. 

## 3. Results

### 3.1. Systematic Review of the Literature and Meta-Analysis

The total number of records identified through database searching was 1271, in addition, records identified through other sources was 13, total records 1284. A total of 15 duplicate records were removed, 1269 total records were screened, and 450 records were excluded. A total of 819 full-test records assessed for eligibility were analyzed. We excluded 779 for following reasons: 82 records because they were case reports/case series, 11 were comments, 10 were editorials, 7 clinical guidelines, 79 were systematic reviews, 13 were meta-analyses that did not include cross-sectional studies on risk factors, 577 were about other topics. A total of 40 papers were considered for systematic review (Figure 1) [4,9,10,11,12,13,15,16,17,18,33,34,35,36,37,38,39,40,41,42,43,44,45,46,47,48,49,50,51,52,53,54,55,56,57,58,59,60,61,62].

The diagnostic test used for screening was ultrasound, except for Denmark where TC scan was used [33].

The selected studies for systematic review and in meta-analysis are summarized in Table 1. Author, year of publication, city or region, age-group, level of participation (%) or screened people (*n*) and AAA detection rate (%) and screening program start were reported. A total of 14 papers were considered in Meta-Analysis, 13 prevalence studies and 1 case-control hospital-based study. 

#### 3.1.1. Gender

**Figure 2:**
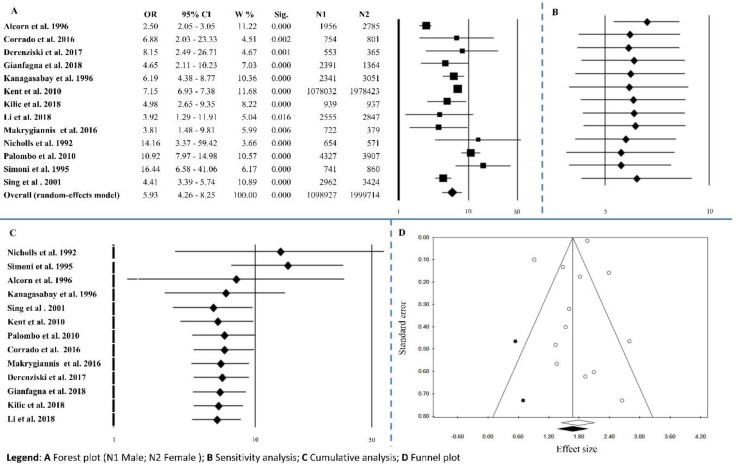
Gender. (**A**) Forest plot (N1 Male; N2 Female); (**B**) sensitivity analysis; (**C**) cumulative analysis; (**D**) funnel plot.

Thirteen studies, among those selected, reported information on males versus females. The overall effect size was OR = 5.93 (4.26–8.25), *p* < 0.0001, with Q = 132.89, *I*^2^ = 90.97, *p* < 0.0001 (Figure 2A and Table 2). Sensitivity analysis shows an equal trend among studies (Figure 2B). Cumulative analysis indicated that all the studies agreed except for Nicholl’s [51] and Simoni’s [36] (Figure 2C). Although publication bias analysis, by the trim and fill method filled two studies (Figure 2D), the results of Egger’s linear regression test and Begg’s and Mazumdar’s rank correlation tests were not statistically significant (*p* = 0.339 and *p* = 0.542, respectively) (Table 2). Meta-regression analysis was not statistically significant *p* = 0.058.

#### 3.1.2. Smoking Habits

**Figure 3:**
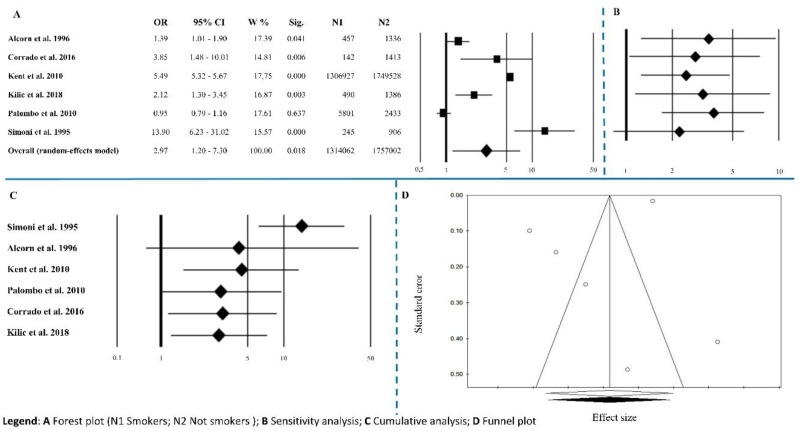
Smoker. (**A**) Forest plot (N1 Smokers; N2 Not smokers); (**B**) sensitivity analysis; (**C**) cumulative analysis; (**D**) funnel plot.

Six papers reported information about smoking habits. The overall effect size was OR = 2.97 (1.20–7.30), *p* = 0.018, with Q = 390.71, *I*^2^ = 98.72, *p* < 0.0001 (Figure 3A and Table 2). Sensitivity analysis showed an unequal trend among studies (Figure 3B). Cumulative analysis indicated that all the studies agreed except for Simoni’s [36] (Figure 3C). Publication bias analysis by the trim and fill method did not involve the exclusion of any papers (0 filled studies) (Figure 3D). The absence of publication bias is underlined by the results of Egger’s linear regression test and Begg’s and Mazumdar’s rank correlation tests that were not statistically significant (*p* = 0.229 and *p* = 0.573, respectively) (Table 2). Meta-regression analysis was not statistically significant *p* = 0.633.

#### 3.1.3. Hypertension

**Figure 4:**
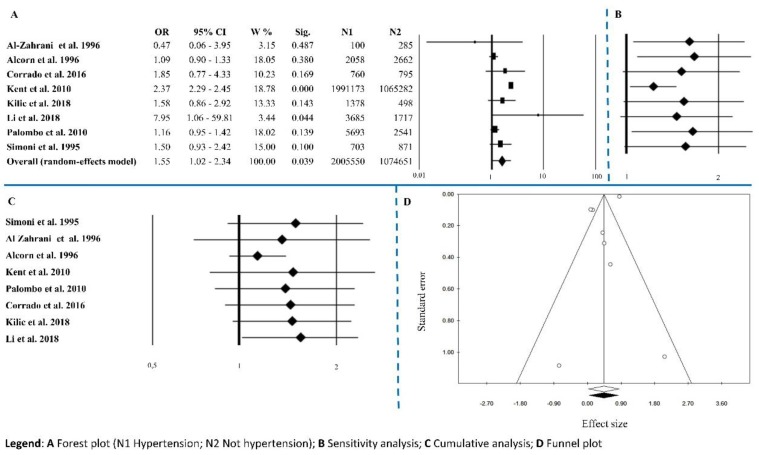
Hypertension. (**A**) Forest plot (N1 Hypertension; N2 Not hypertension); (**B**) sensitivity analysis; (**C**) cumulative analysis; (**D**) funnel plot.

Eight papers contained frequencies on hypertension. The overall effect size was OR = 1.55 (1.02–2.34), *p* = 0.039, with Q = 112.34, *I*^2^ = 93.77, *p* < 0.0001 (Figure 4A and Table 2). Sensitivity analysis showed an equal trend among studies except for Kent’s [55] (Figure 4B). Cumulative analysis indicated that all the studies agreed except for Alcorn’s [57] (Figure 4C). Publication bias analysis by the trim and fill method did not exclude any papers (0 filled studies) (Figure 4D). The absence of publication bias is highlighted from results of Egger’s linear regression test and Begg’s and Mazumdar’s rank correlation tests that were not statistically significant (*p* = 0.127 and *p* = 0.322, respectively) (Table 2). Meta-regression analysis was not statistically significant *p* = 0.202.

#### 3.1.4. Diabetes Mellitus

**Figure 5:**
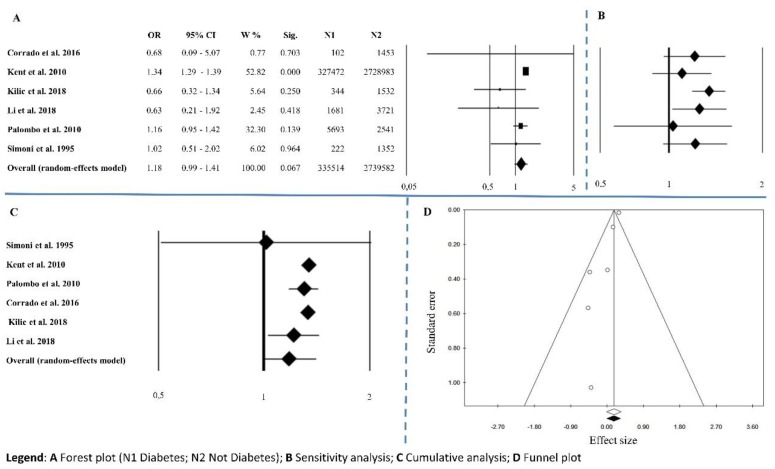
Diabetes. (**A**) Forest plot (N1 Diabetes; N2 Not Diabetes); (**B**) sensitivity analysis; (**C**) cumulative analysis; (**D**) funnel plot.

Six papers reported information on diabetes mellitus. The overall effect size was OR = 1.18 (0.99–1.41), *p* = 0.067, with Q = 8.45, *I*^2^ = 40.85, *p* = 0.133 (Figure 5A and Table 2). Sensitivity analysis showed an unequal trend among studies (Figure 5B). Cumulative analysis indicated that all the studies agreed except for Simoni’s [36] (Figure 5C). Publication bias analysis by the trim and fill method did not involve the exclusion of any papers (0 filled studies) (Figure 5D). According to Egger’s linear regression test (*p* = 0.008) there is bias, but Begg’s and Mazumdar’s rank correlation tests (*p* = 0.851) do not show presence of publication bias (Table 2). 

#### 3.1.5. Coronary Artery Disease

**Figure 6:**
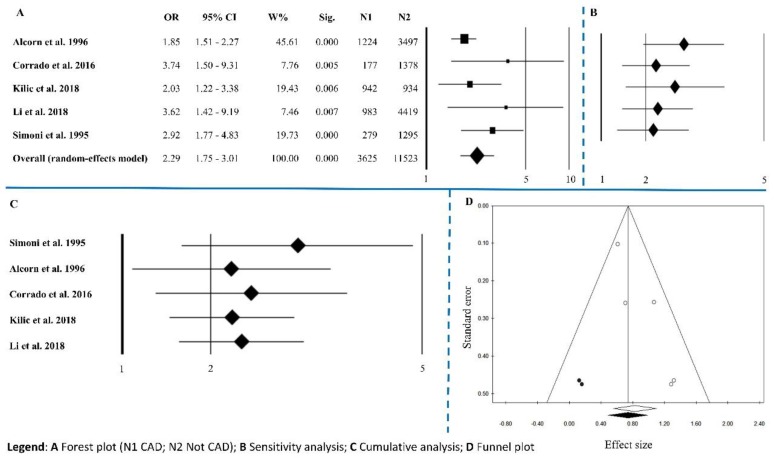
Coronary Artery Diseases (CAD). (**A**) Forest plot (N1 CAD; N2 Not CAD); (**B**) sensitivity analysis; (**C**) cumulative analysis; (**D**) funnel plot.

Information on Coronary Artery Disease was reported in 5 studies. The overall effect size was OR = 2.29 (1.75–3.01), *p* < 0.0001, with Q = 5.98, *I*^2^ = 33.15, *p* = 0.200 (Figure 6A and Table 2). Sensitivity analysis showed an equal trend among studies except for Alcorn’s and Kilic’s [12,57] (Figure 6B). Cumulative analysis indicated that all the studies agreed except for Simoni’s [36] (Figure 6C). Publication bias analysis, by the trim and fill method, filled two studies (Figure 6D). The results of Egger’s linear regression test are statistically significant (*p* = 0.032) but, Begg’s and Mazumdar’s rank correlation tests were not statistically significant (*p* = 0.624) (Table 2). 

#### 3.1.6. Family History of Abdominal Aortic Aneurysm

**Figure 7:**
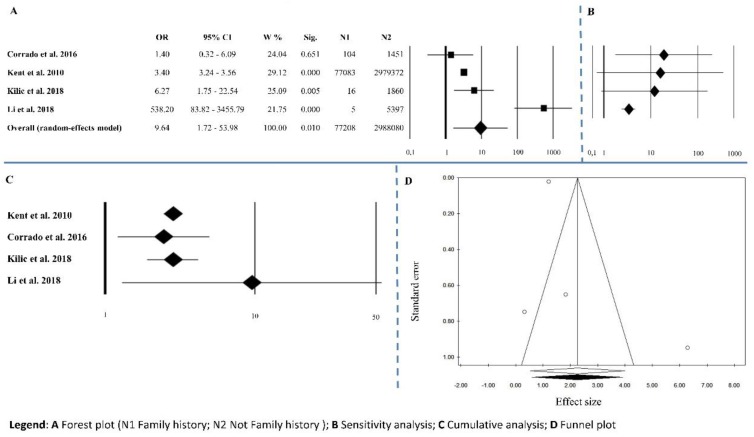
Family history of abdominal aortic aneurysm (AAA). (**A**) Forest plot (N1 Family history; N2 Not Family history); (**B**) sensitivity analysis; (**C**) cumulative analysis; (**D**) funnel plot.

Four studies reported information on family history of AAA. The overall effect size was OR = 9.64 (1.72–53.98), *p* = 0.01, with Q = 30.77, *I*^2^ = 90.25, *p* < 0.0001 (Figure 7A and Table 2). Sensitivity analysis showed an equal trend between studies except for Li’s [15] (Figure 7B). Cumulative analysis indicated that all the studies agreed except for Li’s [15] (Figure 7C). Publication bias analysis by the trim and fill method did not exclude any papers (0 filled studies) (Figure 7D). The absence of publication bias is highlighted from Egger’s linear regression test and Begg’s and Mazumdar’s rank correlation tests that were not statistically significant (*p* = 0.467 and *p* = 0.174, respectively) (Table 2). Meta-regression analysis was not statistically significant *p* = 0.551.

## 4. Discussion

In this study we show the results of a systematic review and a meta-analysis of observational studies. Some Italian studies included in our systematic review have shown AAA prevalence between 1.4% and 6.2% [13,19,35,36]. Other studies showed a range of prevalence AAA from 0.3% to 12.4% or between 0.5% and 9.3% [4,9,10,11,13,18,19,33,51,52,53,54,55,56,57,58,59]. These differences could be due to the different age ranges of the enrolled patients in the studies. A previous meta-analysis on studies about the role of risk factors such as gender, smoking habits, hypertension, diabetes mellitus, myocardial infarction and peripheral vascular disease in development of AAA was conducted in 2004 [64]. We performed a new meta-analysis considering the same risk factors and adding CAD and family history of AAA with more updated studies. In respect to previous meta-analysis [64], we added the funnel plot to examine the effect sizes estimated from individual studies as measure of their precision; sensitivity analysis to check the stability of study findings and estimate how the overall effect size could be modified by removal of one study; cumulative analysis to evaluate the trend between studies in relation to publication year.

We performed a meta-analysis using the random effect according to Der Simonian and Laird for calculate the overall effect-size [65].

Respect to the systematic review, the first important aspect that has emerged is that the organized screening is nationwide only in the UK and Sweden [58,40,41,42,43,44], while in other countries it is mainly local (regional or provincial), as in Italy. In particular, Italy is one of the countries with a substantial number of screening programmes, but most of these are organized mainly in the North (Genoa, Como, Varese) [13,19,35,36]. 

The second important consideration is that only high-income level countries have activated AAA screening programmes as highlighted in cancer screening [66,67]. Therefore, this aspect should be considered in order to avoid social inequalities and greater flexibility for access to treatment and to prevention of AAA. Altobelli et al. [66,67,68,69,70] showed that in many European countries there are no primary prevention campaigns against the main risk factors related to non-communicable diseases and therefore in these nations there is scarce attention to prevention. Sildoff et al. [71] compared the mortality due to AAA in some countries where population-based screening is active, like UK, Sweden, Australia, compared to those where there is no population-based screening, like Austria, Hungary and Romania. In those countries where screening campaigns are active, the mortality rate is in constant decline. The introduction of AAA screening saves lives, prevents rupture risk, coincides with a lower prevalence of the disease, reduces the incidence of aneurysm rupture, and decreases the mortality [1,9,19,35,37,41,58,71,72]. Kim et al. [71] demonstrated that the group invited to be screened had approximately half the risk. The risk reduction was even greater in patients who attended the screening.

Regarding the development of AAA, smoking is the main risk factor correlated to AAA [4,9,10,11,12,13,15,16,17,18,19,33,63,73]. The results of our meta-analysis, relative to male gender and smoking habits, are in line with those of previous research [4,11,12,13,15,19,35,36,45,48,51,55,57]. In our analysis, male smokers have a major risk of AAA. In agreement with some authors [11,12,13,15,19,35,36,48,51,55,57], our results confirm male gender and smoking habits as risk factors for AAA (OR = 5.93 and 2.97, respectively). In countries where the consumption of cigarettes has been reduced, a lower prevalence of AAA has been shown [13,18,19,42,55,59,74]. According to Laroche et al. [18] the reduction in AAA prevalence is parallel to the reduction in tobacco consumption, but anti-smoking information campaigns are insufficient.

Smoking is closely correlated with the diameter of the aorta; it, indeed, is bigger in smokers compared to non-smokers and also according to Al-Zahrani et al. [53] AAA was eight times more in smokers than non-smokers. Therefore, large aneurysm is considered high-risk for rupture and its reduction is essential for reducing aneurysm-related death [54].

Current smoking is associated with occurrence of AAA at younger ages [55]. Moreover, risk of AAA is higher for current smokers than past smokers and it increases with duration of smoking [55].

In the Multicentre Aneurysm Screening Study (MASS) the benefit of quitting smoking has been shown and this benefit leads to decrease of aortic rupture [74].

Some studies that include females show that AAA prevalence is always higher compared to males [4,11,17,18,34,35,39,42,44,47,50,51,54,55]. It is important to underline that association between males and AAA could be attributed to a greater predisposition of males than to females to cardiovascular disease, known as “male disadvantage” [75]. 

According Forsdahl et al. [63] male gender, advancing age, low High-density lipoprotein (HDL) cholesterol and smoking are risk factors associated with AAA and therefore they are factors to be investigated. Other studies have shown an association among AAA and the following diseases: Hypertension, peripheral vascular disease, ischemic heart disease, previous myocardial infarction, chronic obstructive respiratory disease, symptoms of occlusive arterial and coronary artery [44,46,53]. Takei et al. [52] considered risk factors in population target, atherosclerosis, hypertension, obesity, abnormal serum lipid levels and history of smoking. In our meta-analysis hypertension presents an effect size of 1.55 and *p* = 0.039. Regard to the role of hypertension as potential risk factor for AAA some authors [8,37,44,60,63] are in disagreement. Alcorn et al. [57] suggest that individuals with hypertension are more likely to be evaluated clinically for the identification of AAA and this leads to a greater number of AAA diagnoses. Our results show that family history of AAA is also a risk factor, but it is important to underline that family history should be considered with caution because the confidence interval is wide enough, therefore effect size pooled could be influenced.

Respect to gender, smoking habits, hypertension and family history of AAA, the data of our meta-analysis showed presence of heterogeneity. The absence of homogeneity could be due to different sample sizes among studies included in our meta-analysis. Egger linear regression test and mostly Begg’s and Mazumdar’s rank correlation tests show absence of a publication bias. The homogeneity among studies included was supported by Cochrane’s and Higgins’s tests. For such risk factors there is no publication bias. In agreement with previous studies diabetes mellitus is not statistically significant [4,11,12,15,16,19]. De Rango and collegues [76] suppose that high blood glucose forms advanced glycation-end products due to the non-enzymatic oxidation of vascular matrix protein, which over time, becomes less inclined to dilatation and leads to a different sphygmic wave propagation. Our study has some strengths and limitations. The strength of this meta-analysis conducted within the context of a systematic review of descriptive observational studies over a period of 25 years, offers an efficient and potent tool to summarize the clinical evidence accrued on this specific clinical question. Despite its strength, that includes statistical precision and analysis external validity, there are some limitations due to primary studies, which do not allow subgroup analyzes due to lack of data collection; moreover, it is not possible to establish temporal sequence between exposure to risk factors and the onset of the aneurysm because it is a meta-analysis of prevalence studies.

## 5. Conclusions

Abdominal aortic aneurysm is correlated to risk factors associated to an incorrect lifestyle, such as smoking, a wrong diet, absence of regular exercise and gender. Kent et al. [55] found that consumption of fruit, vegetables, nuts and regular exercise reduces the risk of AAA. The importance of a correct diet is also highlighted in other diseases related to nutrition [77,78]. Male gender and family history of AAA are “non-modifiable” factors; while diabetes mellitus, smoking habits, hypertension, CAD can be avoided and, therefore, are “modifiable.” In fact, it is important to underline that quitting smoking, following a correct diet and practicing sports could reduce risk of AAA and consequently the mortality due to rupture of the aorta. 

In addition, our systematic review showed that all countries where AAA screening was set up, were at high income level and the majority belong to Western Europe (United Kingdom, Sweden, Italy, Poland, Spain, Belgium). The purpose of this meta-analysis was to provide a contribution to future research on the role of common risk factors, such as gender, smoking habits, hypertension, CAD, family history of AAA and to address AAA screening to target population at high risk.

The best method of AAA screening is ultrasonography, which is cheap, accurate, safe, rapid, noninvasive, has good reproducibility and is cost-effective. 

In conclusion, these findings, together with continuous lengthening of average life, foreshadow a real “vascular emergency.” Prevention is a fundamental aspect of modern medicine that should be promoted and incentivized in a healthcare system that takes care not only of the illness itself but of the person, even when one is apparently in good health.

## Figures and Tables

**Table 1:** Characteristics of the studies included in the Systematic Review.

Country ^a^ Reference, Year	Region	Age-Group	Level of Participation (%) or Screened People	AAA Detection Rate (%)	Program Start	Included in Meta-Analysis
**Population-Based**						
**Italy**						
Gianfagna, 2018 [13]	Varese, Lombardia	M 50–75 F 60–75	M 65.3 F 61.3 T 63.8	M 1.3 F 0.3 T 0.9	2013	Yes
Palombo, 2010 [35]	Genoa, Liguria	M, F 65–92	M 61.6 F 48.8 T 54.3	M 10.8 F 1.1 T 6.2	2007–2009	Yes
Simoni, 1995 [36]	Genoa, Liguria	M, F 65–75	M 58.5	M 8.8 F 0.6 T 4.4	1991–1994	Yes
**Belgium**						
Makrygiannis, 2016 [4]	Chaudfontaine, Liege, Wallonia	M 65–85 F 74–85	M 39.5 F 31.7 T 36.0	M 4.8 F 1.3 T 3.6	2014	
Vazquez, 1998 [33]	Liege, Wallonia	M 75–65	T 41.0	T 4.5	1995	
**China**						
Kun Li, 2018 [15]	Zhengzhou City, Middle China	M, F <55 M, F 55–75 M, F >75	M 2555 F 2847 T 5402	M 0.55 F 0.14 T 0.33	2014–2015	Yes
**Denmark**						
Dahl, 2018 [61]	Viborg, Central Denmark	F (Born 1936, 1941, 1946, 1951)	F 107,491	NR	2011–2013	
Kvist, 2016 [34]	Northen part of Funen and City of Odense	T 65–74	M 64.9 F 63.0	M 12.4 F 1.1	2014–2015	
**Poland**						
Dereńzíski, 2017 [11]	Gniewkowo, Central Poland	M >60 F >65	M 61.0	M 6.3 F 0.82 T 4.12	2009–2012	yes
Janwien, 2014 [37]	Kuyavia-Pomeranian	M >60	M 1556	M 6.0	2009–2011	
**Spain**						
Sisó-Almirall, 2017 [38]	Barcelona, Catalonia	M 60–65	M 74.9	M 1.5	2013	
Salcedo Jódar, 2014 [9]	Ciudad Real, Castilla La Mancia	M 65–80	M 93.5	M 3.3	2012	
Salvador-González, 2016 [10]	Barcelona, Catalonia	M 65–74	M 66.9	M 2.3	2007	
Barba, 2013 [59]	Asturias	M (born in 1943)	M 70.8	M 4.7	2013	
**Sweden**						
Johansson, 2018 [63]	Uppsala, Dalarna, Södermanland, Västra Götaland	M >65	M 25,265	NR	2006–2009	
Stackelberg, 2017 [40]	Vastmanland, Orebro	M 65–75	M 49.0	M 1.2	2007–2009	
Wanhainen, 2016 [41]	All Nation except Halland Country	M 65–75	M 84.0	M 1.5	2006–2014	
Hager, 2013 [42]	Őstergötland	M >70	M 84.0	M 3.0	2008–2010	
Svensjö, 2013 [43]	Uppsala and Darlana	F >70	M 74.2	F 0.4	2007–2009	
Svensjö, 2011 [44]	Uppsala, Darlana, Sörmland, Gävleborg	M >65	M 85.0	M 1.7	2006–2010	
**United Kingdom**						
Oliver-Williams, 2018 [58]	Gloucestershire, England	M 65	M 80.7	M 1.9	1990–2015	
Kanagasabay, 1996 [45]	London, England	M, F 65–80	NR	M 7.6 F 1.3	1995	Yes
Smith, 1993 [46]	Birmingham, England	M 65–75	M 76.3	T 8.4	1981–1999	
Grismhaw, 1994 [47]	Birmingham, England	M, F 60–75	M 76.1	M 7.2	1989–1991	
**Norway**						
Singh, 2001 [48]	Tromsø	M, F 25–84	25–44 62.0 45–54 81.0 55–64 83.0 65–74 79.0 75–84 58.0	M 9.7 F 2.2 T 4.7	1994–1995	Yes
**Japan**						
Takei, 1995 * [52]	Ueno, Central Japan	M, F 60–79	M 69.0	M 3.9 F 5.0 T 4.6	1992	
**United States**						
Alcorn, 1996 [57]	Pittsburgh cohort	M, F >65	T 656	T 2.9	1990–1992	Yes
**Not Population-Based**						
**Australia**						
Nicholls, 1992 [51]	Perth	M, F 60–80	T 1225	M 4.7 F 0.35 T 2.64	1991	Yes
**Italy**						
Corrado, 2016 [19]	Como, Lombardia	M, F 60–85	T 1555	M 2.5 F 0.4 T 1.4	2010–2013	Yes
**France**						
Laroche, 2015 [18]	All Nation *(metropolitan and overseas departement* *“Operation Vésale”)*	M 50–75 F 60–75	T 6691	M 3.1 F 0.3 T 1.7	2013	
**Greece**						
Makrygiannis, 2018 [60]	Larissa, Central Greece	NR	NR	NR	2010–2013	Yes
**Spain**						
Belloch García, 2018 [16]	La Ribera, Spain	T >50	T 241	T 2.9	2016–2017	
Ortega-Martín, 2007 [39]	León	M 65–75	M 66.0	M 4.2	2000–2001	
**Norway**						
Krohn, 1992 * [49]	Oslo	M, F 60–89	T 500 **	NR	1991	
**Switzerland**						
Engelberger, 2017 [50]	Lugano, Ticino	M 65–80	M 68.2	M 4.1	2013	
**Saudi Arabia**						
Al-Zahrani, 1996 [53]	Jeddah, Western Saudi Arabia	M, F 60–80	NR	T 2.0	1991–1992	Yes
**Turkey**						
Kilic, 2018 [12]	Turkey	T ≥ 65	T 1948	T 3.7	2016–2017	Yes
**United States**						
Chun, 2016 [54]	North Carolina (Veterans Affair Health care system)	M 65–75	T 9571	T 7.1	2007–2011	
Kent, 2010 [55]	All Nation	M, F <85	T 3,056,455	M 1.7 F 0.2 T 0.7	2003–2008	Yes
Lederle, 2000 [56]	15 Department of veterans affair	M, F 50–79	NR	T 1.4	1994–1997	

Legend; NR: Not Reported; E: Echography; M: Male; F: Female; T: Total Sample Size; ^a^ All countries have high income level; * Aorta diameter >2.5 cm; ** The study report only the results of first 500 patients.

**Table 2:** Meta-analysis with studies including male and female.

Risk Factors	Pooled Analysis				Heterogeneity			Publication Bias	
	k = *n*. of Studies	ES (OR)	95% CI	*p*-Value	Q	*p*-Value	*I* ^2^	Egger *p*-Value	Begg and Mazumdar *p*-Value
Gender	13	5.93	4.26–8.25	<0.0001	132.89	<0.0001	90.97	0.339	0.542
Smoking habits	6	2.97	1.20–7.30	0.018	390.71	<0.0001	98.72	0.229	0.573
Hypertension	8	1.55	1.02–2.34	0.039	112.34	<0.0001	93.77	0.127	0.322
Diabetes mellitus	6	1.18	0.99–1.41	0.067	8.45	0.133	40.85	0.008	0.851
Coronary Artery Disease (CAD)	5	2.29	1.75–3.01	<0.0001	5.98	0.200	33.15	0.032	0.624
Family history of AAA	4	9.64	1.72–53.98	0.01	30.77	<0.0001	90.25	0.467	0.174
